# Mothers with schizophrenia: Treatment, quality of life and motherhood experience

**DOI:** 10.1192/j.eurpsy.2021.1375

**Published:** 2021-08-13

**Authors:** S. Pérez Sánchez, I. Martín Herrero, M.A. Cutillas Fernández, A. Crespo Portero, D. Raya Güimil

**Affiliations:** 1 Servicio De Psiquiatría, Servicio Murciano de Salud, Murcia, Spain; 2 Servicio De Psiquiatría, Servicio Murciano de Salud, Lorca, Murcia, Spain

**Keywords:** Schizophrenia, mother, paliperidone palmitate, quality of life.

## Abstract

**Introduction:**

Schizophrenia is a chronic disease that deteriorates the functionality of patients, especially when forming a family and taking care of children. We are interested in analyzing the characteristics of mothers with schizophrenia and their degree of global activity when going from oral treatments to injectable treatments.

**Objectives:**

1 To assess the quality of life and functional level of mothers with schizophrenia receiving paliperidone treatment. 2. Compare quality of life and functional level when going from oral treatment to long-term injectables.

**Methods:**

Sample: Mothers, 37-45 years old, diagnosed with schizophrenia in monotherapy with oral paliperidone who started treatment with Paliperidone Palmitate LD IM (200 - 300 mg / month). Retrospective data collection. QLS quality of life scale.

**Results:**

5 patients were included, caregivers of 1 child (80%), 2 children (20%) who met the inclusion criteria and completed the questionnaires. After its application and correction through non-parametric tests (N <30). During oral treatment, scores were observed in the QLS questionnaire of: mean intrapsychic functions 34.2, mean interpersonal relationships 19, mean instrumental role 8, mean daily activities 8. After 12 weeks of treatment with Paliperidone Palmitate IM, scores were obtained: functions Medium intrapsychic 36, medium interpersonal relationships 23, medium instrumental role 15, medium daily activities 11. A better functioning of the patients was observed in the instrumental and daily activities categories.

**Conclusions:**

In our experience, injectable long-acting Paliperidone Palmitate is associated with the perception of better quality of life in mothers with schizophrenia and increases the ease of administration as well as planning in their daily life.

